# Proceedings from the Third International Post-Tuberculosis Symposium: expanding the circle

**DOI:** 10.5588/ijtldopen.25.0637

**Published:** 2025-12-10

**Authors:** B.W. Allwood, S.C. Auld, B. Beko, G.P. Bisson, C.P. Borges de Almeida, A. Byrne, F.C. Chow, A. Davis, S. Defres, M. Drage, D. Evans, X. Gai, G. Günther, A.N. Gupte, G. Hoddinott, M.A. Huaman, S. Huddart, J. Huynh, G. Kalyatanda, C. Khosa, T. Kutadza, G. Makanda, S. Marais, J. Meghji, N. Navuluri, E. Nkereuwem, A. Rajaratnam, K. Romanowski, I. Schoeman, J.A. Seddon, H. Sohn, F. Thienemann, D.T. Wademan, N.F. Walker, M.M. van der Zalm, R. Nightingale

**Affiliations:** 1 Division of Pulmonology, Department of Medicine, Stellenbosch University and Tygerberg Hospital, Cape Town, South Africa;; 2 Departments of Medicine, Epidemiology and Global Health, School of Medicine and Rollins School of Public Health, Emory University, Atlanta, GA, USA;; 3 TB Proof, Cape Town, South Africa;; 4 Department of Medicine, Perelman School of Medicine at the University of Pennsylvania, Philadelphia, PA, USA;; 5 Faculty of Public Health, Federal University of Sul e Sudeste do Pará, Marabá, Brazil;; 6 Department of Thoracic Medicine, St Vincent’s Hospital and Clinical School, University of New South Wales, Sydney, NSW, Australia;; 7 Socios En Salud Sucursal, Lima, Peru;; 8 Departments of Neurology and Medicine, Weill Institute for Neurosciences, University of California San Francisco, San Francisco, CA, USA;; 9 The Francis Crick Institute, London, UK;; 10 Wellcome Centre for Infectious Diseases Research in Africa, Institute of Infectious Disease and Molecular Medicine, University of Cape Town, Cape Town, South Africa;; 11 Queen Mary and Barts Tuberculosis Centre, Queen Mary University London, London, UK;; 12 Tropical and Infectious Diseases Unit, Liverpool University Hospitals Foundation NHS Trust, Liverpool, UK;; 13 LIV-TB group and Department of Clinical Sciences, Liverpool School of Tropical Medicine, Liverpool, UK;; 14 LHL International Tuberculosis Foundation, Oslo, Norway;; 15 Health Economics and Epidemiology Research Office, Faculty of Health Sciences, University of the Witwatersrand, Johannesburg, South Africa;; 16 Department of Respiratory and Critical Care Medicine, Peking University Third Hospital, Beijing, China;; 17 Department of Pulmonology, Allergology and Clinical Immunology, Inselspital, Bern University Hospital, University of Bern, Bern, Switzerland;; 18 Boston University School of Public Health, Boston, MA, USA;; 19 Desmond Tutu TB Centre, Department of Paediatrics and Child Health, Faculty of Medicine and Health Sciences, Stellenbosch University, Cape Town, South Africa;; 20 Department of Internal Medicine, University of Cincinnati College of Medicine, Cincinnati, OH, USA;; 21 Department of Epidemiology and Biostatistics, School of Medicine, University of California San Francisco, San Francisco, CA, USA;; 22 Oxford University Clinical Research Unit, Ho Chi Minh City, Vietnam;; 23 Centre for Tropical Medicine and Global Health, Nuffield Department of Medicine, Oxford University, Oxford, UK;; 24 Division of Infectious Disease and Global Medicine, Department of Medicine, University of Florida College of Medicine, Gainesville, FL, USA;; 25 Instituto Nacional de Saúde, Marracuene, Mozambique and Department of Physiological Sciences, Clinical Pharmacology, Faculty of Medicine, Universidade Eduardo Mondlane, Maputo, Mozambique;; 26 Departments of International Public Health and Clinical Sciences, Liverpool School of Tropical Medicine, Liverpool, UK;; 27 Zimbabwe National Network of PLHIV, Harare, Zimbabwe;; 28 Division of Neurology, Department of Medicine and Neurology Research Group, Neuroscience Institute, University of Cape Town, Cape Town, South Africa;; 29 National Heart and Lung Institute, Imperial College London, London, UK;; 30 Department of Medicine, Duke University School of Medicine and Duke Global Health Institute, Durham, NC, USA;; 31 Vaccines and Immunity Theme, Medical Research Council Unit, The Gambia at London School of Hygiene and Tropical Medicine, Banjul, The Gambia;; 32 Faculty of Infectious and Tropical Diseases, London School of Hygiene and Tropical Medicine, London, UK;; 33 Department of Epidemiology, Rollins School of Public Health, Emory University, Atlanta, GA, USA;; 34 Department of Global and Public Health, McGill University, Montreal, QC, Canada;; 35 Department of Infectious Disease, Imperial College London, London, UK;; 36 Department of Preventive Medicine, Seoul National University College of Medicine, Seoul, Republic of Korea;; 37 General Medicine and Global Health Research Unit, Department of Medicine, University of Cape, Cape Town, South Africa;; 38 University of Zürich, Zürich, Switzerland;; 39 Centre for Tuberculosis Research and Department of Clinical Sciences, Liverpool School of Tropical Medicine, Liverpool, UK;; 40 Department of Respiratory Medicine, Liverpool University Hospitals Foundation Trust, Liverpool, UK.

**Keywords:** tuberculosis, post-TB lung health, sequelae, advocacy

## Abstract

In light of the recent growth in interest and knowledge of post-TB sequelae, there were high levels of engagement during the 3rd International Post-Tuberculosis Symposium held in Stellenbosch, South Africa. This multi-disciplinary symposium aimed to: 1) Advocate for greater global awareness of post-TB sequelae and empower TB-affected communities; 2) Advance knowledge by sharing current evidence and identifying key priorities; 3) Foster collaborations by strengthening research networks and developing concrete plans for research driven advocacy; and 4) Advance the field by establishing areas of consensus around diagnosis, care, and management. Guided by a 14-member Steering Committee, 9 academic working groups came together to develop key content for plenary sessions and facilitated workshops related to: Patient Engagement, Epidemiology and Modelling, Pathogenesis, Post-TB Lung Disease; Cardiovascular and Pulmonary Vascular Disease; Central Nervous System and Musculoskeletal Disease; Paediatrics Economic; Social and Psychological Sequelae; and Advocacy, Policy, and Stakeholder Engagement. Each group outlined progress within their respective fields and defined key priorities to focus discussion. The Symposium further catalysed coordinated action for the post-TB community of patients, advocates, clinicians, and researchers to define a clear path towards improving outcomes, reducing inequities, and ensuring TB survivors receive the care and support they deserve.

Since the 1st International Post-Tuberculosis Symposium was held in 2019,^1^ there has been exponential growth in the interest and knowledge around post-TB sequelae, which was also reflected in conference engagement and attendance. The 3rd International Post-Tuberculosis Symposium was held in Stellenbosch, South Africa, from 14 to 16 April 2025. This multi-disciplinary meeting, dedicated entirely to life after TB, was led by a 14-member steering committee with patient advocacy and global representation. The Symposium aims were:

1. Advocacy – To increase global awareness of post-TB sequelae and empower TB-affected communities.
2. Knowledge – To update knowledge on post-TB life and illness and identify research priorities.
3. Networking – To build research collaborations and set up concrete plans for research advocacy.
4. Advancement and Consensus – To identify areas for consensus around diagnosis, care, and management and establish roadmaps for achieving consensus.

The Symposium covered nine thematic areas and consisted of both state-of-the-art presentations and facilitated workshops. There were 224 registered delegates, including TB survivors, in attendance, representing 134 institutions from 31 countries (6 continents). The 9 working groups involved more than 100 individuals who came together prior to the Symposium to develop content. This document provides a summary of the Symposium proceedings, with the goal of further expanding awareness and knowledge about post-TB disease, burden, and ongoing efforts to ameliorate post-TB sequelae. Of note, Symposium sessions are available for viewing online at https://www.post-tuberculosis.com/.

## PATIENT ENGAGEMENT WORKING GROUP

The Symposium emphasised the critical role of patient engagement in addressing the ongoing impact of TB on survivors’ lives. Sixteen TB survivors attended the Symposium in person, representing diverse experiences including living with HIV, TB during pregnancy, TB meningitis, and adolescent TB. TB survivors from four high-burden countries – Cambodia, Indonesia, India, and South Africa – shared their experiences either in person or through recorded interviews that were presented during the plenary sessions.

An Indonesian TB survivor living with HIV spoke about their experience with stigma:

I didn’t have the courage to open up to my family at that time. The double-stigma made me hesitant to speak openly, fearing that I would be seen as having a bad family background.

The survivor further described how their recovery from TB was limited by a lack of access to information, which underscored the multifaceted challenges of stigma and the need for accessible information in TB care and recovery.

A young woman from India spoke about the difficulties she experienced after completing TB treatment in 2017. She stated:

I was TB cured in 2017, but that was not the end of it. […] When my [medical] reports came, it came out that my left lung [had] completely collapsed. This whole time, I fought TB only to realise that TB had permanently damaged my body.

The young woman was later diagnosed with axonal neuropathy, a form of nerve damage that can arise from either TB or its treatment and result in both sensory and motor dysfunction. She described how TB rendered her unable to ‘live a normal life’, severely impeding her ability to study, work, and support her family. She described the constant fear of recurrence, the significant psychological impact of the disease, and concluded that:

Life after TB, and the complications that come with it, like mine, are real and life-long.

A TB survivor from Cambodia highlighted how TB can impact families and wider communities, with five of her family members affected over the years. Her father's illness, the first in the family, forced her to drop out of school to help support her family. Since becoming a TB advocate, she has used her powerful voice to inform her community about TB symptoms and services, demonstrating the potential for survivor-led health education. A TB survivor from South Africa presented the final TB survivor plenary session, delivering a moving talk about her multiple TB recurrences and their impact on her mental, physical, and financial well-being. She concluded her talk by encouraging all people affected by TB to persist with treatment and to hold onto hope for a better life.

The Symposium’s closing session featured a unique contribution from an 18-year-old TB survivor, who performed a rap song. The young man lamented the impact TB had on his schooling, friendships, and budding music career:

When I’m all alone, all I think about is money;

Woke up this past year because TB did me dirty; Will I ever heal, I don’t know; All this pain in my body’s got to go; […]

TB blew my body apart, yea; I was broken, I was broke, yea.

The rap concluded with encouragement to other young people affected by TB to remember that the road is long, with him serving as proof that a difficult journey can lead to recovery.

## EPIDEMIOLOGY AND MODELLING WORKING GROUP

A central theme from the epidemiology session was the need for epidemiological approaches that could illuminate the heterogeneity observed in respiratory, psychological, and social post-TB sequelae and guide tailored interventions. The session emphasised that aligning research questions with the appropriate analytical approach was critical.^2^ Descriptive analyses were highlighted as a way to characterise the distribution of outcomes and how they differed across populations or contexts. Predictive approaches were discussed as tools to identify who was most likely to experience adverse sequelae, while causal inference methods were presented as essential for determining whether and how TB itself contributed to these outcomes.^2,3^ Clarifying whether the question is descriptive, causal, or predictive is essential for ensuring analyses are well aligned and findings are relevant. The session also emphasised the substantial heterogeneity in post-TB outcomes. While some individuals recovered with minimal lasting effects, others experienced chronic symptoms, functional limitations, or reduced quality of life.^4^ These divergent trajectories reflected the complex interplay of disease severity, treatment response, co-morbidities, and underlying social and structural determinants.^5^ Epidemiological approaches were discussed as critical tools for exploring this diversity and guiding more tailored care strategies.

To better capture this heterogeneity, speakers noted the importance of moving beyond population averages. Trajectory modelling using longitudinal lung function data had been used to identify subgroups with distinct recovery patterns, each with different implications for care.^6^ When longitudinal data are unavailable, other techniques such as latent class analysis or unsupervised clustering were described as useful for defining subgroups based on cross-sectional characteristics.^7^ Even stratification by demographic or clinical features can yield important insights into how outcomes varied across populations. Causal inference tools were also presented as playing a critical role in separating the lasting impacts of TB from pre-existing conditions and broader social influences. Without accounting for confounding factors, it was noted that estimates of TB’s long-term effects might be misleading.^8^ Techniques such as directed acyclic graphs, propensity score matching, and quasi-experimental designs were highlighted as approaches that could strengthen causal conclusions in observational data.^9,10^

Finally, the session underscored the need for greater standardisation. Without consistent definitions, outcome measures, and assessment time points, it is difficult to compare studies or synthesise evidence across settings. Harmonising tools and reporting standards will be key to building a robust evidence base and informing policy. Ultimately, the session concluded that strong epidemiological analysis depended not only on methodological rigour but also on high-quality, detailed data. Incorporating clinical, social, and environmental variables is critical for generating meaningful insights. By pairing the right questions with the right tools, epidemiology can play a powerful role in shaping effective, equitable post-TB care.

## PATHOGENESIS WORKING GROUP

The Pathogenesis Working Group aimed 1) to inform post-TB pathogenesis by learning from a clinical perspective and 2) to feature and highlight ongoing research addressing current gaps in our understanding. This followed work synthesising knowledge on the pathogenesis of post-TB lung disease (PTLD) during the 2nd Symposium.^11^ One of the key themes that emerged from the clinically oriented overview was that the morphology of PTLD is highly variable, and therefore heterogeneity in the pathways and mediators involved in pathophysiology should be expected and anticipated. For example, a histopathological review of surgical specimens of human lung tissue showed multiple granuloma phenotypes, even within a single specimen from an individual patient. Persistent inflammation, fibrosis, and airway remodelling were seen, as well as diverse vascular pathology, reflecting the diversity of mechanisms and pathways driving these changes. At the same time, metaplastic transitions and the presence of tertiary lymphoid structures were relatively consistent findings across post-TB pulmonary specimens. Likewise, following identification of heterogeneous host inflammatory responses to *Mycobacterium tuberculosis* and *M. africanum*, an in vitro model has been developed, informed by an analysis of *M. tuberculosis* complex (MTBC) lineages controlled by host monocytes (unpublished work), to help evaluate candidate host–directed therapeutics (HDT) in clinical trials for the prevention of PTLD.^12,13^ The existence of hypo- and hyperinflammatory subtypes of TB, with different post-TB endotypes,^14^ is consistent with work from other respiratory diseases, from asthma to acute respiratory distress syndrome (ARDS), which have raised awareness of the need for targeted treatments that address the heterogeneity underlying these clinical syndromes.

Highlights of ongoing research on post-TB pathogenesis from across the translational spectrum were presented in interactive sessions in four broad categories: 1) prediction and risk factors, 2) interventions, 3) host–pathogen interactions, and 4) models and morphology. Within prediction and risk factors, recent work from diverse cohorts has identified associations between baseline radiography, complete blood cell count features, neutrophilic biomarkers, and blood transcriptomic signatures and the development and manifestations of PTLD. Interventions to reduce PTLD focused on host-directed therapies, including plans for a study of adjunctive doxycycline to prevent post-TB fibrosis and *ex vivo* assessment of immunomodulatory activity of statin therapy.^15,16^ A need for appropriate, consensus endpoints for clinical trials of host-directed therapies to prevent PTLD was highlighted. Featured host–pathogen interactions addressed the implications for host lung tissue damage based on variable mechanisms of cell death, host metabolism of iron and other metals, and diversity of MTBC strains. Finally, several models for enhancing our understanding of the pathogenesis of PTLD were reviewed, including murine models of comorbid diabetes mellitus and TB-associated fibrosis, and modelling the role of matrix stiffness on mycobacterial growth.

Recent years have led to an encouraging expansion of post-TB pathogenesis research, with insights into risk factors and predictors, as well as progress along the translational spectrum.

## POST-TB LUNG DISEASE WORKING GROUP

In preparation for the symposium, the PTLD working group reviewed the proceedings and outputs from the prior symposia and discussed ongoing challenges in the areas of PTLD research, clinical care, and programmatic implementation. The working group identified one major challenge in each of the three areas, with the goal of identifying strategies to address these challenges through workshops and a consensus-building process (Table 1). Recently published data relevant to these challenges were summarised, and progress in the development of a research definition for PTLD was shared.

**Table 1.** PTLD working group key challenges and approach to solution.

Challenge	Approach to solution
Programmatic: National TB programmes lack guidance on how individuals treated for TB should be evaluated for PTLD	Identify assessments, priorities, and challenges around end-of-treatment PTLD screening to inform future consensus or guideline development
Research: Investigators lack guidance on a core set of clinically meaningful pulmonary endpoints to include in TB clinical trials	Agree on a minimum set of PTLD-related endpoints for TB clinical trials
Individual Care: Clinicians at the frontlines of PTLD care lack tools to help prioritise the investigation and management of PTLD given its complexity and heterogeneity.	Identify core treatable traits (pulmonary, extra-pulmonary, and behavioural/risk factor) for PTLD

### Summary of data relevant to PTLD challenges

Key reviews from the past 3 years were summarised, including those focused on post-TB spirometry, other respiratory sequelae, national TB programme (NTP) guidelines, and interventions to mitigate or manage post-TB respiratory morbidity.^17–21^ Only seven NTP guidelines currently mention PTLD, with wide variation in their recommendations on the investigation or management of PTLD.^22^ A major issue in PTLD assessment is ruling out recurrent TB. The low positive predictive value of DNA molecular tests for active TB in people with prior TB was reviewed.^23^ Data on the TB molecular bacterial load assay, which targets *M. tuberculosis* RNA, were presented, suggesting utility in distinguishing active versus prior TB.^24^ This assay may be useful in distinguishing patients in PTLD studies who need sputum culture.

### Updates on PTLD research definition

The need for a research definition of PTLD to support evidence synthesis and harmonisation of data collection was discussed at the 2nd symposium and further developed with regular meetings. Challenges include a lack of data linking symptoms and signs of respiratory sequelae to long-term clinical outcomes, uncertain cut-points for disease in continuous measures (e.g., lung function), and a lack of validated standards for assessing symptoms and imaging. A draft definition requiring abnormalities in two of three domains of respiratory symptoms, lung function, and radiographic abnormalities was presented and endorsed by attendees.

### Developing programmatic approaches to end-of-treatment PTLD screening

There are no international consensus guidelines about how individuals with TB should be evaluated for residual respiratory morbidity,^5^ and diverse screening approaches have been proposed and used.^25^ This workshop explored feasible approaches to routine PTLD screening within NTPs. End-of-treatment was selected as a practical timepoint for screening that aligns with the timing of documenting TB treatment outcomes.

The working group recognised the drawbacks, from a patient perspective, of screening for PTLD in the absence of clear pathways to post-TB care. Thus, the goal was to work towards consensus on assessments that could identify individuals or populations with either high ongoing morbidity or increased risk for long-term complications for future implementation research. Country-level case studies from Brazil, India, Kenya, Vietnam, and Zambia were used to discuss the potential populations to be screened, types of assessments which could be used for screening (e.g., symptoms, imaging, spirometry, and/or functional assessments), the relative priority of those assessments, and factors influencing prioritisation (e.g., reproducibility, sensitivity, specificity, cost, ease of interpretation, and correlation with outcomes). A panel discussion explored the benefits and challenges of end-of-treatment screening and the need for standardised approaches. Among a survey of 80 workshop participants, 96% agreed that NTPs should implement some form of end-of-treatment assessments for PTLD and 91% felt that at least some aspects of these assessments should be standardised across countries.

### Establishing PTLD clinical trial outcomes

This workshop aimed to identify a core set of pulmonary outcomes to be included in TB clinical trials (e.g., new TB treatment regimens, TB vaccines, and host-directed therapies), to evaluate the burden of PTLD in the setting of different interventions and support standardised reporting and evidence synthesis. An overview of HDT trial endpoints and a case study of key considerations and challenges in selecting appropriate pulmonary endpoints were used to open discussions. Small groups discussed five outcome ‘domains’ of pulmonary function, functional status, patient-reported outcomes, lung imaging, and health care utilisation using five questions: 1) Should this domain be used as an outcome measure in TB clinical trials? 2) If yes, how should it be measured? 3) When should it be measured? 4) What constitutes a clinically meaningful difference in the selected measure/parameter? and 5) Are there surrogate endpoints within each domain that should be considered?

### A consensus approach to identifying core PTLD ‘treatable traits’ to guide clinical care

A treatable traits approach has proved effective in the management of several chronic respiratory diseases with heterogeneous phenotypes and pathogenesis, including asthma, chronic obstructive pulmonary disease, and bronchiectasis.^26,27^ This approach requires the identification of specific traits that are clinically relevant, identifiable or measurable, and associated with adverse outcomes or co-morbidities, and for which effective and acceptable treatments are available. A first survey round exploring the potential for a treatable traits approach in PTLD focused on symposium attendees’ current approach to the investigation and management of PTLD. Of 155 respondents, 94.2% agreed that the current approach to management of PTLD in their setting could be improved. The second survey round presented a list of potential treatable traits that were either pulmonary, extra-pulmonary, or related to behaviours or broader risk factors for PTLD disease. In the third survey round, workshop participants narrowed the list of potential post-TB treatable traits to the most important and relevant in three categories: pulmonary (10 traits), extra-pulmonary (5 traits), and behavioural/risk factors (5 traits) based on five different priorities: i) patient perspective, ii) treatability, iii) impact and risk, iv) connected comorbidity, and v) prevalence.

## CARDIOVASCULAR AND PULMONARY VASCULAR WORKING GROUP

Discussion centred on the epidemiology, mechanisms, and clinical implications of post-TB cardiovascular disease (CVD). Existing observational data consistently indicate that TB increases the risk of CVD.^28–30^ Overall, it is estimated that the risk of CVD events in people with TB is at least 1.5 times higher than control populations without a history of previous TB.^31^ Recent evidence indicates that the risk of CVD may be the greatest within 3 months of the TB diagnosis, but excess CVD morbidity persists even after successful TB treatment (post-TB phase, duration unclear).^32^ Although studies have primarily focused on atherosclerotic CVD (i.e., myocardial infarction, stroke, and peripheral arterial disease), emerging data suggest blood pressure and other metabolic syndrome parameters can also be altered during and after TB treatment.^33^ Additionally, there is increasing recognition of TB as a risk factor for venous thromboembolism with studies indicating 2-3 times increased risk of deep vein thrombosis and pulmonary embolism events before and during the TB treatment.^34^ Ongoing studies are investigating the effects of TB and associated lung pathology on overall cardiac function, the myocardium, and pericardium.

There are several potential mechanisms through which TB may increase cardiovascular risk.^35^ Traditional cardiovascular risk factors such as tobacco use, diabetes mellitus, hyperlipidaemia, and hypertension are common in individuals with TB and likely contribute to post-TB CVD morbidity and mortality. At cellular and molecular levels, TB induces pathological processes that can lead to CVD, including epigenetic scars, immunosenescence, mitochondrial dysfunction, lipid peroxidation, and hypercoagulation.^35–37^ It is unknown whether certain TB drugs, environmental factors such as air pollution, and pathogen characteristics such as *M. tuberculosis* lineage and virulence could influence CVD risk in TB, which needs further study.

Cardiovascular risk stratification and modifiable risk factor assessment and intervention are recommended in the general population. Routine use of available CVD risk prediction calculators is encouraged in people with TB and post-TB to identify those individuals who would benefit the most from evidence-based primary CVD prevention strategies.^37^ Appropriate management of tobacco use, diabetes mellitus, hypertension, and hyperlipidaemia based on regional or international guidelines is expected to have substantial impact on CVD prevention in TB and post-TB. Encouraging healthy lifestyle modification could be implemented in routine care of individuals with TB and post-TB, whereas pharmacologic and more specialised management may require coordination with care teams outside TB programmes. During discussion, it was recognised that there is a need to better integrate TB and HIV and non-communicable disease (NCD) services; however, the best way to implement such integration requires investigation.^38^

The symposium also included discussion on potential biomarkers and non-invasive methods for measuring vascular function and predicting vascular morbidity post-TB. There was recognition that research in this field needs to be cross-disciplinary and should encourage involvement from vascular physiologists and cardiologists, among others. Illustrative cases of post-TB haemoptysis highlighted the complex pathophysiology, clinical presentation, and challenging management of a common and potentially life-threatening pulmonary vascular complication after TB.

## CENTRAL NERVOUS SYSTEM AND MUSCULOSKELETAL WORKING GROUP

Discussion focused on the long-term impact of central nervous system (CNS) TB, which includes TB meningitis (TBM), the most common manifestation of CNS infection, along with CNS tuberculomas and spinal TB, commonly known as Pott’s disease. TBM is regarded as the most lethal form of TB and frequently results in long-term disability. Residual functional impairment after completion of CNS TB treatment remains poorly characterised but includes motor and sensory deficits, seizures, cognitive impairment, hearing and vision loss, and psychiatric and behavioural manifestations.^39^

### Defining post-CNS TB and evaluation of patients

Workshop discussion centred around a research definition as well as the composition and timing of follow-up assessments, that could be used to assist the development of strategies in support of health care systems and to standardise research approaches aimed at improving CNS TB outcomes. Use of a framework that considers how CNS TB affects day-to-day function, activity, and the ability for survivors to participate in family life, work, and community was highlighted as essential in the development of a definition for post-CNS TBM disability.

### Determining research priorities for post-CNS TB

A discussion of research priorities in post-CNS TB focused on areas to improve clinical outcomes. Emphasis was placed on the need for a better understanding of the negative health consequences, and socio-economic impact on patients and families. Research is needed to identify modifiable risk factors for poor outcomes, host-directed therapies to improve outcomes, and use of scoring tools for predicting clinical disease and outcome. An important theme highlighted by survivors was the need for community-based research in affected populations. Investigations into community perceptions of the disease, barriers to care, stigma and false beliefs surrounding lumbar puncture, and the disease itself were priorities raised by survivors to improve mental and physical outcomes of those affected.

### Establishing meaningful outcomes from a patient perspective

Survivors, caregivers, and advocates contributed richly to a focus group describing the lived experiences of those affected by CNS TB. Patients described diverse sequelae ranging from reduced mobility, vision impairment, and debilitating fatigue to emotional lability affecting social reintegration. Functional outcomes (e.g., ability to self-care, mobilise, play with others, learn and attend school, and having normal family interactions) featured prominently among child survivors. In adults, returning to independent employment and mental health was important. These insights highlight the heterogeneity and long-lasting impacts of CNS TB, underscoring the importance of tailoring care and support based on outcomes most valued by survivors and caregivers and the need to integrate these into future clinical research.

### Exploring clinical priorities, long-term goals, and how to get there

A crucial aspect of post-CNS TB care was the integral role of community health workers and peer advocates. There was a strong emphasis on developing more robust peer advocacy programmes to help navigate treatments for sequelae and life after hospital discharge. Patients, caregivers, and health care workers all require easily accessible education and resources on a range of topics related to long-term functional capacity (e.g., physical and occupational therapy exercises).

## PAEDIATRIC WORKING GROUP

The paediatric post-TB working group focused on TB-associated respiratory morbidity across childhood and adolescence. Since the previous Symposium in 2023, the evidence base has expanded, with six new studies published, underscoring growing interest in PTLD in children.^40–45^ Emerging data show that respiratory impairment is common among children and adolescents following TB treatment, with the burden increasing with age.^46^ In children under 5 years of age, impairments such as reduced tidal volume and peak expiratory flow have been observed. Among those aged 5–10 years, approximately 40% show abnormal lung function post-treatment, and this figure increases to 65% in adolescents over 10 years of age. Additionally, between 35% and 50% of children and adolescents report ongoing respiratory symptoms. Children under 10 years also show reduced growth metrics and impaired quality of life.

TB presents with age-specific patterns, varying from lymph node and disseminated disease in infants to adult-type pulmonary TB in adolescents.^47^ The impact of TB, particularly its age-specific disease features, on the developing lung and its implications for future lung capacity and regenerative potential remain poorly understood.^48^ The group emphasised the complex and multidimensional impact of TB and its treatment, including issues of stigma and mental health challenges, on children’s overall well-being.^49^ The long-term physical, psychological, and social outcomes associated with PTLD are also not well-explored.^50^

To address these gaps, the working group identified the need to develop standardised approaches, including the harmonisation of tools, definitions, and outcome measures, to enable robust cross-study comparisons. The working group session was structured around the expertise required for developing these standards, including epidemiological, health economic, and lung function expertise. Development of such standards will help advance the field and is essential for informing early interventions, policy development, and resource planning for the growing population of childhood and adolescent TB survivors. This work is planned as a follow-up to the symposium, with the goal of establishing a collaborative framework for standard development.

## ECONOMIC, SOCIAL, AND PSYCHOLOGICAL WORKING GROUP

The economic, social, and psychological (ESP) working group has had growing interest over the years, which has paralleled a notable increase in research outputs in this space. However, this growth has highlighted the methodological challenges of delineating ESP sequelae, as each overarching domain – economic, social, and psychological – encompasses multiple interrelated sub-components. These challenges are further compounded by heterogeneity in how sequelae manifest by setting, population, disease episode severity and trajectory, and the limited availability of social protections for people affected by TB. The lack of standardised quantitative tools and variability in qualitative data dramatically limit the ability to compare across contexts and slow the growth of a cohesive body of evidence to inform policy and practice for ESP health during the post-TB period.

**Figure 1.**
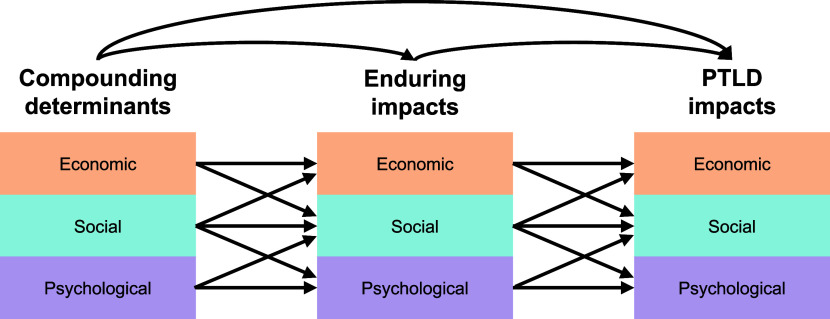
Preliminary version for a conceptual model for ESP-associated post-TB sequelae.

Underlying these challenges are the fundamental questions of what constitutes post-TB ESP sequelae and how they can be consistently defined across settings. Equally critical is determining how to collect comparable data across diverse contexts and populations, including establishing clear parameters for measurement timing and priority outcomes to assess (Table 2). The ESP working group engaged the first stage of a Delphi process towards consensus on the scope of ESP measures and a working conceptual model of the ESP-associated sequelae of TB (Table 2). The working group also agreed to maintain a clear distinction between ‘social’ and ‘psychological’ sequelae, preserving them as separate domains. The links between ESP-related consequences of post-TB sequelae and the ESP-associated upstream drivers of TB risk are poorly understood. At the workshop, we presented a preliminary version of the conceptual model that highlights the complexity of ESP-associated post-TB sequelae (Figure 1).

**Table 2.** ESP-associated post-TB sequelae.

	Economic	Social	Psychological
ESP-associated post-TB sequelae	• Missed education (children and adolescents)	• Quality of life	• Children – cognitive and emotional development, memory, play
	• Missed employment opportunities	• Stigma	• Mental health issues, including anxiety and depression
	• Cost burdens to the family	• Peer disclosure	• Self-esteem and self-worth
	• Credit owed (borrowed/loan + interest)	• Resilience	• Loss of relationships (intimacy, friendships, etc.)
	• Income loss and job loss	• Participation in social groups/clubs	• Fear of relapse
	• Labour market participation and earnings	• Loss of group membership	• Quality of life – physical and mental well-being
	• Household income and expenditure	• Nutrition and food security	• Post-traumatic stress disorder
	• Assets accumulation and erosion	• Social role	
	• Human capital accumulation	• Equity	
	• Social protection uptake	• Impact on relationships	
	• Direct and indirect costs		
	• Shocks and stresses		
	• In-kind support		
	• Costs of ‘maintaining face’		
	• Gender and generational costs		
	• Encumbered career progression/promotion		
	• Low productivity (hours worked)		
	• Catastrophic costs		
	• Employment/unemployable		
	• Cumulative costs to households of >1 episode across the household		

During the facilitated workshops, three parallel breakaway sessions brought together clinicians, researchers, policymakers, and TB survivors to share lived experiences across the economic, social, and psychosocial dimensions; key discussion points from each are summarised here.

In the economic domain, the group outlined current evidence on the financial impact of post-TB sequelae and TB recurrence. The group also discussed recent findings from Korea,^51^ Canada,^52^ the United States, and the United Kingdom^32^ that show TB patients experience considerably higher levels of health care utilisation post-TB relative to those who did not have TB.

In the social domain, we engaged examples of how stigma intersects with both psychological and economic consequences. In discussion, the group noted the shared impression that men and women are often differently affected by TB-related stigma and that TB frequently disrupts personal relationships, affecting couple and family dynamics. Interventions should promote education, psychosocial support, resilience, and attitude change across individuals and the community.

In the psychological domain, the group noted that there may be significant delays in the onset of the psychological impacts and considered the viability of extrapolating from our knowledge around post-traumatic stress disorder to the psychological effects of TB. The group noted that the psychological impacts of a TB disease episode often manifest in interpersonal consequences and interventions must focus on the relational experiences of TB. We identified a key concern around the psychological impacts of post-TB sequelae, as TB in early life can directly cause lasting emotional distress and stigma, while indirect effects, like parental stress and strained relationships, further compound the burden, contributing to long-term psychosocial, health, and educational challenges.^53^

We identified five key priorities: 1) generation of primary data on ESP outcomes (see Table 2) and patient costs during and after TB treatment; 2) assessment of the cost of illness attributable to TB disease as new data and analytic approaches emerge; 3) advancement of conceptual, multi-disciplinary, and mixed-method research; 4) development of targeted interventions; and 5) evaluation of the effectiveness of interventions aimed at preventing and mitigating post-TB ESP sequelae and supporting long-term TB survivorship.

## ADVOCACY, POLICY, AND STAKEHOLDER ENGAGEMENT WORKING GROUP

### Prioritising post-TB care on the national and international health agenda

Many individuals who complete TB treatment continue to face physical, psychological, social, and economic challenges. Evidence-based recommendations to support people with post-TB disease are available, but often not prioritised at the country level. At the Symposium, collective advocacy priorities for post-TB care were set based on country-level pilot projects, scientific evidence, and perspectives from TB survivors and affected communities.

### Person-centred post-TB care should be integrated in national TB recovery plans and national TB strategic plans, including treatment algorithms and post-TB indicators

Individuals with persistent lung impairment, symptoms, or psychosocial challenges are often unable to access care or support or may be unable to afford the services or treatments when available. National TB strategies must move beyond cure to include comprehensive, person-centred care that promotes long-term recovery. Embedding accessible post-TB support into policy frameworks is essential to ensure survivors are not left behind in the care continuum.^54^ With South Africa’s National TB Programme Manager leading the way at the Symposium, there was consensus that post-TB care should be implemented as a priority, and that:

Post-TB care should be available to increase the quality of life of TB survivors. The Department of Health should add a post-TB indicator to monitor individuals needing post-TB care, with urgent health worker training on how to provide such care.

Civil society organisations have a key role to create demand for post-TB care and empower TB-affected communities.

### Implementation considerations: pilot projects demonstrating feasibility, impact, and scalability

The power of data-driven advocacy has been proven in Malawi and Tanzania, where pulmonary rehabilitation (PR) programmes at community and district levels are now integrated as part of the national TB strategies in both countries.^55^ Advocacy with a multi-sectoral approach is key at the very beginning, strengthening the linkage between health systems and community. Each PR site should have a close link with a health facility where a focal person is assigned to make regular contact and visit the site. Our recommendations for continuing to scale-up such PR programmes are: 1) Bring the intervention as close to the people as possible to overcome accessibility barriers (e.g., transportation); 2) Partner with civil society and TB survivors (peers) for implementation and training; 3) Collect data to show impact and benefits; and 4) Integrate post-TB PR programmes into the national TB strategy.

**Figure 2.**
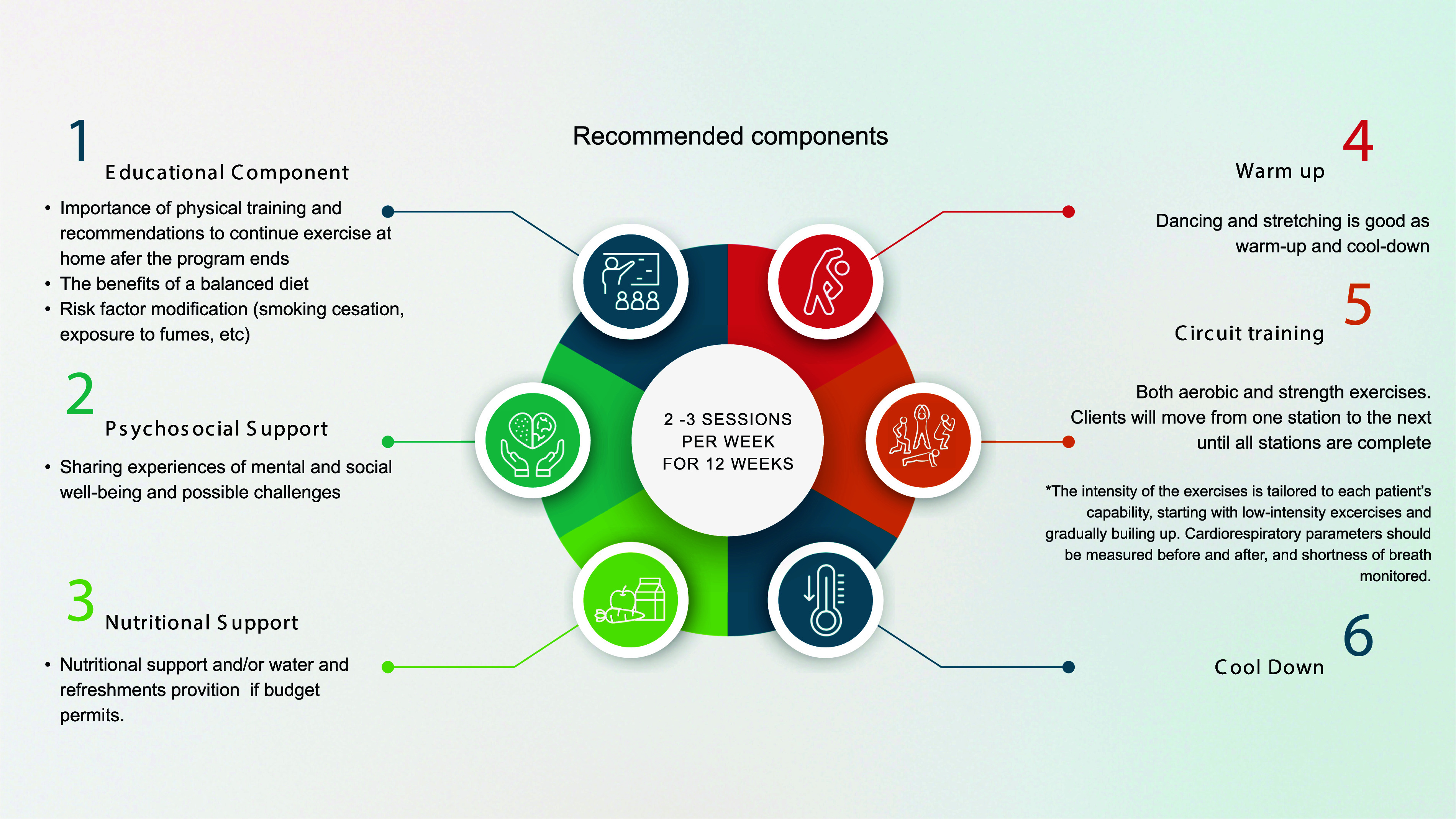
Recommended components for establishing a low-cost pulmonary rehabilitation programme.

PR service packages typically include breathing exercises, strength and endurance training, counselling, nutritional support, social support, and health education. Since PR programmes include a physical examination, participants can be screened and referred for NCD care as appropriate (e.g., based on elevated blood pressure) (see Figure 2). Current evidence suggests that PR for at least 12 weeks may increase exercise capacity, reduce dyspnoea (breathlessness), improve quality of life and mental health, reclaim daily function, and reintegrate into society faster.^56^ Programmatic data also suggest sustained effects in exercise capacity, respiratory health, and mental well-being being at least 1 year after PR completion.^57^ Further data on the effectiveness and timing of PR for PTLD are eagerly awaited.^58^

For policy makers, PR programmes may be considered relatively low-cost and scalable interventions, especially when community- or home-based models are employed. Furthermore, decentralised services can improve access in rural or underserved areas. By improving self-management and reducing complications, PR has the potential to lower long-term health care costs; healthier individuals are likely more economically productive, theoretically reducing indirect costs related to disability or unemployment. However, data are urgently needed to support these empiric assumptions about cost-effectiveness and long-term societal benefits.

## CONCLUSION

The 3rd International Post-Tuberculosis Symposium was another pivotal step towards improved advocacy, knowledge sharing, networking, and advancement in response to the now well-recognised long-term consequences of TB. Framed by powerful testimonies of TB survivors, the multi-disciplinary working groups presented ongoing research and took important steps in working towards proposed frameworks for standardising definitions, endpoints, and care strategies, as well as advocating for the programmatic implementation of post-TB care. In many thematic domains (e.g., PTLD), these steps form the building blocks that will move the field towards effective interventions that will prevent and treat post-TB sequelae. However, knowledge and implementation gaps remain for multiple domains, including paediatrics and cardiovascular and neurological sequelae, and patient-centred advocacy remains critical to defining research priority areas.

Through the efforts and advocacy of the Symposium Community, awareness and interest in the field of post-TB consequences has been increasing exponentially, with Symposium attendance doubling with each iteration. After an open voting process at the 3rd Symposium, the 4th International Post-Tuberculosis Symposium will take place in Seoul, Republic of Korea in 2027. The 4th Symposium will continue to support efforts to increase global advocacy, which will be facilitated by hosting the Symposium in Asia, a high-TB-burden geographical region.

For the estimated 155 million TB survivors alive globally,^59^ and the millions more who develop TB each year, identifying, preventing, and treating post-TB sequelae is an urgent health care priority, which demands coordinated action from clinicians, researchers, policymakers, and affected communities. The 3rd International Post-Tuberculosis Symposium was an important catalyst in coordinating action against this real, complex, and far-reaching global health problem, and defining a clearer path to improve outcomes, reduce inequities, and ensure TB survivors receive the care, dignity, and support they deserve.
